# Microscopic evidence for nanoparticle-mediated growth of native gold in sulfide deposits at the Higashi–Aogashima Knoll Caldera hydrothermal field

**DOI:** 10.1371/journal.pone.0317220

**Published:** 2025-01-17

**Authors:** Satoshi Okada, Junji Torimoto, Takahiro Kuribayashi, Toshiro Nagase, Akira Owada, Jun-ichiro Ishibashi, Akiko Makabe, Yutaro Takaya, Tatsuo Nozaki

**Affiliations:** 1 Institute for Extra-cutting-edge Science and Technology Avant-garde Research (X-star), Japan Agency for Marine-Earth Science and Technology (JAMSTEC), Yokosuka, Japan; 2 Faculty of Science and Engineering, Waseda University, Tokyo, Japan; 3 Department of Earth Science, Tohoku University, Sendai, Japan; 4 The Center for Academic Resources and Archives, The Tohoku University Museum, Tohoku University, Sendai, Japan; 5 Nippon Petrographic Thin Section Co., Ltd., Inashiki, Japan; 6 Kobe-Ocean Bottom Exploration Center, Kobe University, Kobe, Japan; 7 Department of Systems Innovation, The University of Tokyo, Tokyo, Japan; 8 Submarine Resources Research Center, Japan Agency for Marine-Earth Science and Technology, Yokosuka, Japan; 9 Frontier Research Center for Energy and Resources, The University of Tokyo, Tokyo, Japan; K Ramakrishnan College of Technology, INDIA

## Abstract

Gold (or electrum) in hydrothermal fluid precipitates directly from gold sulfide complex and/or partly via suspended nanoparticles. The hydrothermal fluid contains “invisible gold” that is atomically dispersed in sulfide minerals or as nanoparticles with a size of less than 10 nm. However, the contribution of these gold nanoparticles to the formation of native gold and its alloy with silver (electrum) remains unclear. The Higashi–Aogashima Knoll Caldera hydrothermal field, south of Tokyo, Japan, is an area of significant seafloor hydrothermal activity that is known for high-grade gold-containing minerals in sulfide-rich rocks. In this study, dry-polished thin sections were created to minimize sample damage and scanning and transmission electron microscopy were used to investigated the cross-sectional and three-dimensional morphologies of native gold grains in a sulfide-rich mound rock from the Central Cone site of the caldera. The surfaces of the gold grains comprised nanoparticles with sizes of 5–50 nm that were also attached to their periphery, which suggests that gold nanoparticles in deep-sea hydrothermal fluid were involved in the mineralization of the gold. In addition, the distribution of silver was uneven within the gold grains, which suggests that the gold precipitation comprised multiple stages at different temperatures that resulted in the post-deposition or secondary remobilization of silver.

## Introduction

Sulfide deposits formed by hydrothermal activity on the seafloor are an important mineral resource that can potentially be mined in the future for metals such as copper, lead, zinc, gold (Au) and silver (Ag) [1–3]. Some of these deposits are associated with arc volcanoes and are located at relatively shallow depths of less than 2,000 m below sea level (mbsl). These deposits may provide key evidence for hydrothermal fluid boiling below the seafloor [4, 5], which is an efficient mechanism for precipitating noble metals such as Au from their atomically dissolved forms such as [Au(HS)_2_]^−^ in the form of colloidal particles [6–8]. Colloidal gold nanoparticles (NPs) approximately 10 nm in size are associated with arsenian pyrite [9, 10] and have been identified in crystalline gold plates [11] while larger submicron particles have been discovered in fluid inclusions of quartz from subaerial epithermal Au–Ag deposits [12] and in black smoker discharge from hydrothermal vents [13, 14]. Electrum is an alloy of Au and Ag especially when the Au and Ag concentrations are 20–80 wt% [15], and is also found in the form of colloidal NPs with diameters of 2–20 nm in amorphous carbon inclusions formed in the voids of coarse gold grains [16]. These gold or electrum NPs are considered forms of “invisible” gold, along with Au dispersed atomically in the matrix of sulfide minerals [17, 18]. The presence of these NPs in both the voids of minerals and the venting hydrothermal fluids has led to the assumption that gold or electrum mineralization proceeds directly from hydrothermal fluid [19] or via a two-step process mediated by gold NPs, depending on the supersaturation and fluid’s physicochemical conditions of the fluid [13, 14]. However, the process by which these NPs accumulate and form large grains in deep-seafloor hydrothermal deposits has been difficult to visualize.

**Fig 1:**
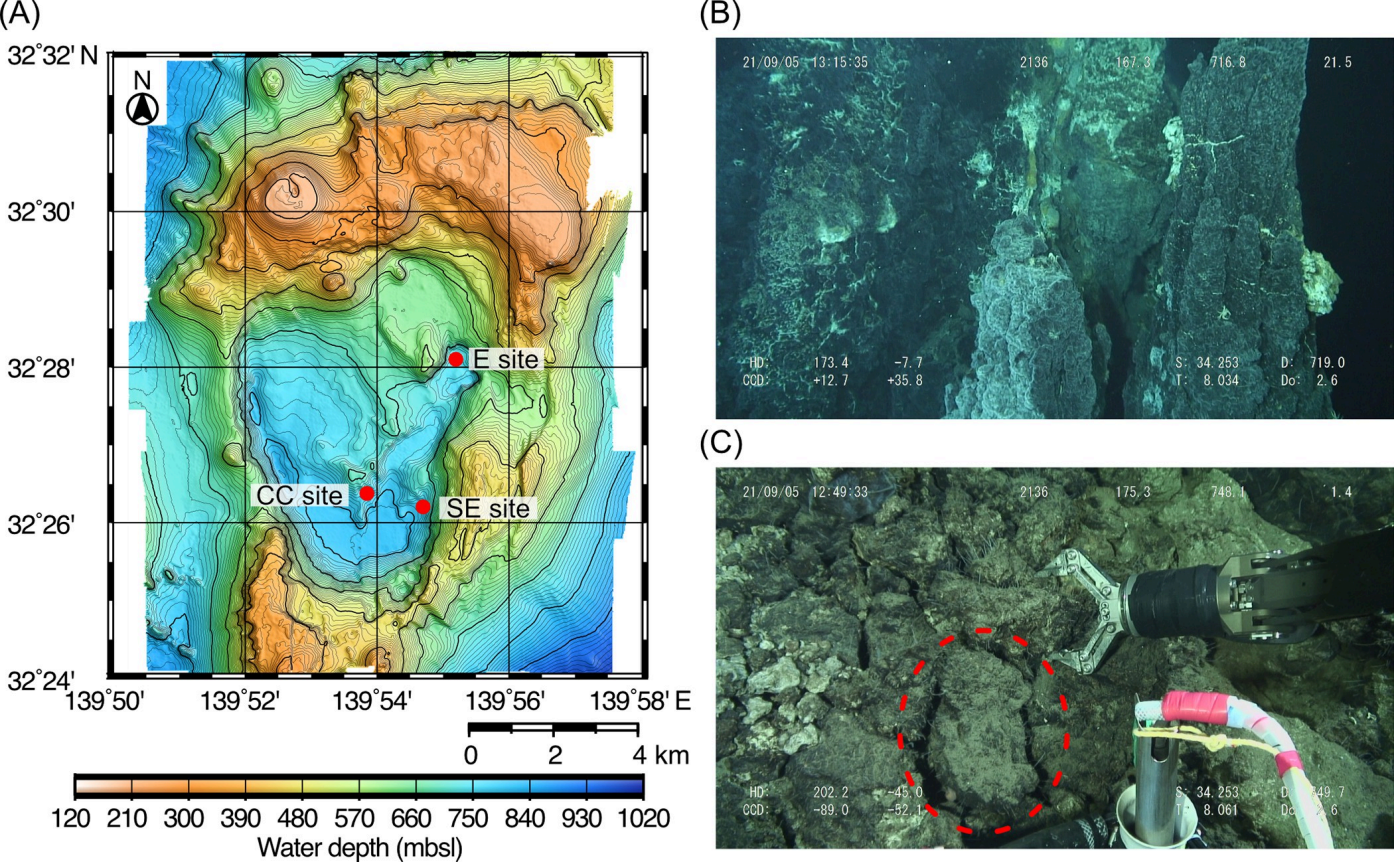
Sample collection. (A) Locations of the CC, SE, and E sites at the HAKC HF. Bathymetric data were obtained by *R/V Yokosuka* during cruise YK21-10 [26] using a Kongsberg EM122 multi-beam echo sounder at a frequency of 12 kHz. The map was drawn by using the open-source software Generic Mapping Tools Version 6 [27]. (B) Photo of a chimney swarm at the CC site taken during a dive. (C) Photo of the northern flank of a sulfide mound at the CC site taken during a dive. The red circle indicates the sample used in this study.

In 2015, the Higashi–Aogashima Knoll Caldera (HAKC) seafloor hydrothermal field (HF) was discovered along the Izu-Ogasawara volcanic front within Japan’s exclusive economic zone (Fig 1A) [20–22]. The HAKC HF is located at ~750 mbsl and the Central Cone site has sulfide mounds with average Au and Ag concentrations of 102 and 432 ppm, respectively (*N* = 15) [20]. Despite the social awareness of these kinds of deep-sea hydrothermal “gold mines” as future metal resources, the nanoscale morphology and the growth mechanisms of gold remained unclear. In this study, dry polishing [23] and gallium focused ion beam (FIB) serial sectioning [24] were used to expose gold grains in taken from a sulfide mound of HAKC HF, and surface and cross-sectional scanning electron microscopy (SEM) and transmission electron microscopy (TEM) imaging were used to investigate the mechanisms by which optically invisible electrum NPs grew into optically visible native gold grains.

## Sample and methods

The HAKC HF is located 12 km east of Aogashima Island in the Northwestern Pacific. It contains three known hydrothermal sites: the Central Cone (CC), Southeast (SE), and East (E) sites (Fig 1A) [20]. In 2021, the multidisciplinary research cruise expedition KS-21-20 was conducted using the R/V Shinsei-maru and remotely operated vehicle (ROV) Hyper Dolphin (HPD) to investigate the mechanisms by which Au was enriched at the HAKC HF. During this expedition, 30 mound rock samples were collected (Fig 1B and 1C) [25]. For this study, sample HPD#2136R05 retrieved from the northern flank of the sulfide mound at the CC site (Fig 1C; 32°26.3726’N, 139°53.8512’E, 748 mbsl) was selected based on its high bulk Au and Ag concentrations. Polarized optical microscopy of wet-polished section showed that the selected rock sample primarily comprised sphalerite and barite with galena, chalcopyrite, minor amounts of pyrite, As-S minerals (orpiment and realgar), tetrahedrite, enargite, electrum or native gold (indistinguishable by optical appearance), trace amounts of pyragyrite and proustite, as well as gangue amorphous SiO_2_ exhibiting very weak birefringence under crossed-Nicols (opal) and unidentified clay minerals (S1 Fig). The electrum/ native gold grains were predominantly found within sphalerite close to their grain boundaries and around the periphery of sphalerite adjacent to opal (S1 Fig).

During the research expedition, the HPD ROV KS-21-20 conducted dives and took four samples of hydrothermal fluid from active hydrothermal vents and four seawater at ~5 m above the seafloor using bag fluid samplers and Niskin samplers. The temperatures of the hydrothermal fluid and seawater were measured *in situ* by a pressure-resistant thermometer helped by the ROV’s manipulator and a built-in conductivity temperature depth profiler on the ROV’s forehead, respectively. In the laboratory, the pH of the water was measured at room temperature by using a Radiometer PHC2401-8 Combination pH Electrode (Hach). The Cl concentration was determined by titration with silver nitrate solution (Mohr’s method). The maximum temperature, pH, and Cl concentrations were 242.3°C, 4.55–4.74, 588–592 mM at the CC site; 280.4°C, 3.86, 731 mM at the SE site; 271.6°C, 6.15, 557 mM at the E site; and 7.5–8.0°C, 7.56–7.92, and 551–557 mM in seawater, respectively. Note that these are raw values of the hydrothermal fluid after mixing with ambient seawater and do not represent the end-member fluid compositions. The elevated Cl concentration in all hydrothermal fluid samples compared to the ambient seawater suggest that subseafloor boiling (i.e. phase separation) takes place at the HAKC HF, which is a common process for many HFs in the Okinawa Trough and Izu-Bonin arc [28, 29].

The mound rock sample HPD#2136R05 was cut and sliced, rinsed with tap water, and dried. The wet polishing method was used to prepare sections where a block was embedded in a low-viscosity epoxy resin (EpoFix, Struers), polished using diamond plates (roughness: 70, 30, and 13 mm) with water as a lubricant, and then polished using diamond powder (roughness: 6 and 1 mm) on a silk polishing cloth (MD-Dur, Strues) with alcoholic blue lubricant (Strues). The dry-polishing method was also used to prepare sections where a block was cut and sliced without any lubricants, embedded in low-viscosity epoxy resin (CaldoFix-2, Struers), and polished at less than 50 rpm by using a waterproof abrasive paper (SiC foil; roughness: 200, 120, 80, 68, 52, 28, 18, 15, 13, 10, and 5 mm) and alumina abrasive powder (roughness: 1 μm) on a silk polishing cloth (MD-Dur) without any water or other lubricants. The wet- and dry-polished sections were examined under a stereomicroscope (Nikon Eclipse 50i POL) and were then coated with carbon (~20 nm, CADE, Meiwafosys) to facilitate observation by electron microscopy.

The Au and Ag concentrations in sample HPD#2136R05 were measured by inductively coupled plasma quadrupole mass spectrometry (ICP-QMS; Agilent 7500ce, Agilent Technologies, USA) conducted at JAMSTEC in accordance with a previously described method [30]. All reagents for ICP-QMS were TAMAPURE-AA-10 or TAMAPURE-AA-100 grade and were procured from Tama Chemical Co., Ltd. First, 50 mg of a powdered sample was digested by 0.8 mL HClO_4_, 2 mL HF, and 4 mL HNO_3_ at 110°C for 24 h. Then, the sample was dried by stepwise heating (110 → 130 → 160 → 190 °C), after which it was further digested by 3.75 mL HNO_3_, 1.25 mL HCl, and 5 mL Milli-Q deionized water at 110°C to stabilize Au and Ag in the mixed acid solution. This sample was then appropriately diluted for ICP-QMS analysis.

The mineral composition was determined by an electron probe microanalyzer (EPMA: JXA-8500, JEOL) at JAMSTEC equipped with five wavelength-dispersive X-ray spectroscopy (WDS) detectors (XM-86030 and XM-86010, JEOL) operated at an acceleration voltage of 20 kV and a beam current of 12 nA. The incident electron beam was focused to a diameter of 100 nm, and the counting time was 50 s for each element. Natural mineral samples and synthetic metal samples (ASTIMEX MINM25-53, METM25-44) were used as standards. At least three grains of optically visible representative minerals were analyzed (S1 Table). The X-ray lines measured were S, Mn, Fe, Co, Ni, Cu, and Zn (Kα), Ga, Ge, As, Se, Ag, Cd, Sb, and Te (Lα), and Pb and Bi (Mα) for minerals other than opal and Na, Mg, Al, Si, P, S, K, Ca, Ti, Cr, Mn, and Fe (Kα), and As (Lα) for opal. The acquired X-ray intensities were corrected by the atomic number-absorption-fluorescence (ZAF) method [31] and the elemental fractions were recorded as percentages of the weight and atoms for minerals and as percentages of the weight as oxides for opal.

SEM images were acquired by using Helios G4 UX (Thermo Fisher Scientific) at JAMSTEC equipped with a field-emission electron gun, a gallium FIB gun, and an energy-dispersive X-ray analysis (EDS) detector (Octane Elite Super, AMETEK). Secondary electron (SE) and backscattered electron (BSE) images were acquired at an acceleration voltage of 2 or 5 kV and a beam current of 0.1–0.8 nA, and EDS elemental maps were acquired at an acceleration voltage of 20 kV and beam current of 0.8 nA. The EDS elemental maps were recorded on the software TeamEDS software (v. 4.6, AMETEK) for 34 and 55 groups of particles in 14 and 47 EDS maps from wet- and dry-polished thin sections, respectively. Gold grains less than 5 μm in size were considered the same group because they may be cross-sections of a branched gold grain (discussed later). The Au contents of a gold grain was calculated from the weight fraction of the gold phases automatically assigned by the software as [Au]_w_/ ([Au]_w_ + [Ag]_w_), where [X]_w_ denotes the weight fraction of X. The recorded images were processed in Fiji [32] and Affinity Photo 2 (Serif). After SEM imaging and EDS analysis, the sample was coated with a 150-nm-thick layer of carbon (CADE, Meiwafosys) for FIB-SEM serial sectioning. Images were collected by using the software Auto Slice & View (version 4.0.3.846, Thermo Fisher Scientific) at an acceleration voltage of 3 kV, a beam current of 0.8 nA, and a slice pitch of 20 nm. The three-dimensional (3D) images were then reconstructed on the software Amira 2019.2 software (Thermo Fisher Scientific).

TEM samples were prepared by using the FIB-SEM. A protective platinum (Pt) layer was deposited onto gold grain in the dry-polished section, and a lamella with a thickness of 2–4 μm was picked by using a tungsten needle. The lamellar was welded with Pt onto a copper lift-out grid and was thinned to <200 nm in thickness using a Ga ion beam operated at 30 kV followed by 5 kV. TEM images operated at 200 kV were acquired on Tecnai G2 20 (Thermo Fisher Scientific) at JAMSTEC equipped with a bottom-mounted 2k × 2k pixel charge-coupled device (CCD) camera (Eagle, Thermo Fisher Scientific), a side-mounted 1.4k × 1k pixel CCD camera for diffraction (Megaview III, SIS), and EDS detector (RTEM-S 61700ME, AMETEK). The recorded images were processed in Fiji [32] and Affinity Photo 2 (Serif).

## Results

### Surface morphology and composition of gold grains

**Fig 2:**
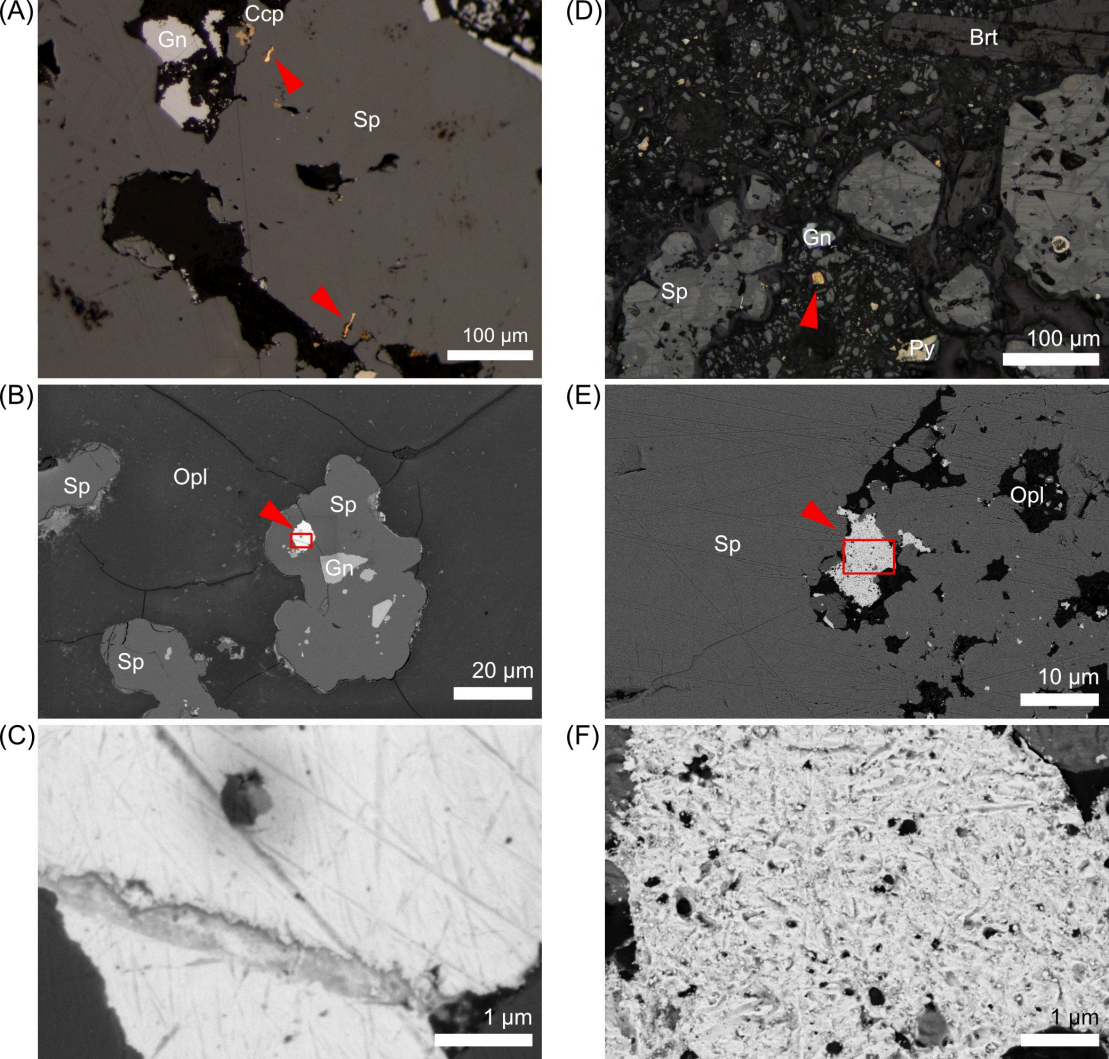
Polished surface of the sulfide samples. (A) Reflected-light photomicrograph, (B) BSE-SEM image, and (C) expanded view of the wet-polished section (red box in B). (D) Reflected-light photomicrograph, (E) BSE-SEM image, and (E) expanded view of the dry-polished section (red box in E). Red triangles indicate gold grains. SEM images were taken at (B, C) 2 kV and (E, F) 5 kV. Abbreviations: Brt, barite; Ccp, chalcopyrite; Gn, galena; Opl, opal; Py, pyrite; Sp, sphalerite.

The ICP-QMS analysis indicated that the HAKC mound rock sample contained zinc, lead, iron, copper, and barium as the major elements (>0.5 wt%) with Ag and Au concentrations of 309 and 179 ppm, respectively. The wet-polished sections showed that the gold grains were yellow-gold in color with a metallic luster under reflected light (Fig 2A). These grains were predominantly in contact with adjacent sphalerite and opal (Fig 2B and S2 Fig). The straight scratches in random orientations were artifacts of the polishing process (Fig 2C). Laser abrasion inductively coupled plasma mass spectrometry (LA-ICP-MS) analysis revealed that the sphalerite was poor in iron with a range of 0.07%–0.48% and average ± standard deviation of 0.28% ± 0.07% (*N* = 60) [33]. SEM-EDS revealed that Ag was mostly found with arsenic and antimony in the forms of pyrargyrite and proustite, which was consistent with optical observations. Ag sulfide was also found without arsenic and antimony, tentatively assigned as acanthite owing to their small size (< 5 μm), which hampered precise quantification by EPMA (S2 Fig). SEM/EDS mapping showed that the gold grains comprised 93.9 ± 4.1 wt% Au (*N* = 14), which indicated that they could be mineralogically classified as native gold.

A distinct difference in morphology was observed for the gold grains in the sections obtained by dry polishing, which is a method used to minimize damage to fragile and ductile materials [23]. In the dry-polished sections, the gold grains had an orange hue with dark spots and a less pronounced metallic luster (Fig 2D). The surface was rougher with numerous dark spots about 100 nm in size and less distinct scratch patterns compared to the wet-polished sections (Fig 2E and 2F). These surface dark spots were attributed to artificial holes formed during the polishing process (Text A in S1 Text). The SEM-EDS analysis showed that among the 55 gold grains observed, 42 grains were in contact with sphalerite while 31 grains were in contact with opal. Only 12 grains were in contact with chalcopyrite and seven grains were in contact with galena while no grains were adjacent to barite. The gold grains were 91.0 ± 3.3% Au, which was equivalent to that of the wet-polished sections. The part of gold grains facing opal, a void, or a grain boundary of sphalerite tended to have a higher concentration of Ag than inside or facing sphalerite (34 grains, S3 and S4 Figs). Many gold grains in the dry-polished samples were adjacent to open voids (unfilled by resin, 35 out of 55 grains, S3 Fig). Residue of alumina abrasive powder (231 ± 85 nm in diameter, *N* = 299) and cutting dust (33 ± 10 nm in diameter, *N* = 61) was detected within these voids and is likely to be an artifact of the dry-polishing process (S4 Fig).

### Three-dimensional morphology

**Fig 3:**
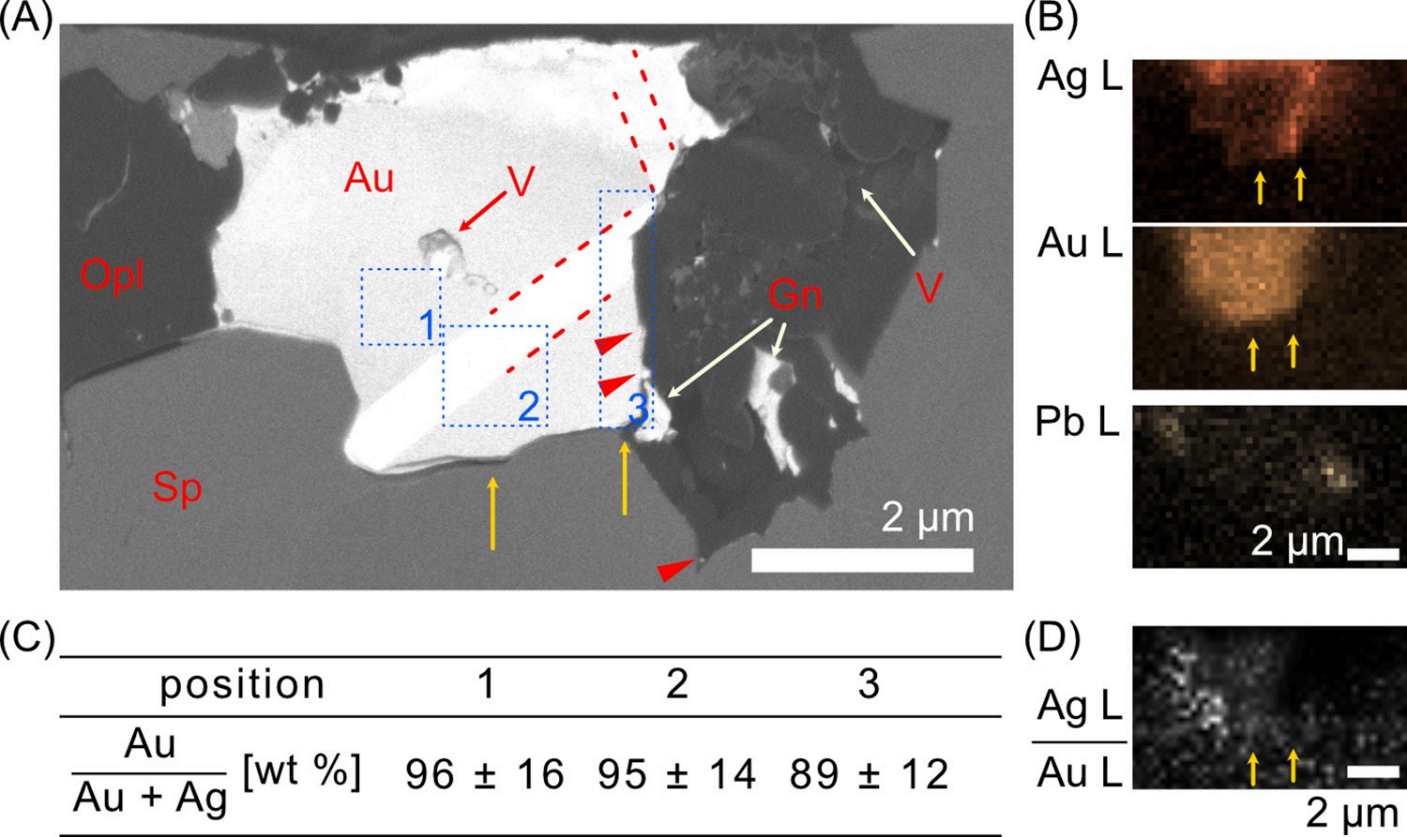
FIB cross-sections of a gold grain obtained from a dry-polished thin section. (A) Cross-sectional image of Fig 2E. Red dashed lines indicate the boundaries of clear contrast differences within the grain. Red triangles indicate gold NPs on the grain. (B) EDS elemental maps of the same region shown in A. Yellow arrows indicate the relative positions of Ag-rich and Ag-poor domains. (C) Au content of the blue dotted square area in (A). (D) Ag L EDS intensity map divided by Au L intensity map. SEM image at 5 kV and EDS analysis at 20 kV. Mineral abbreviations are as in Fig 2 (Au: gold; V: void).

The cross-sections obtained by FIB were used to examine morphology of the gold grains (Fig 3A). The mineral phases were classified based on the difference in relative contrast of the secondary electron (SE) and backscattered electron (BSE) images (Fig A–C in S5 Fig) and assigned by extrapolation of the cross-sectional EDS images (Fig 3B). Gold and galena had similar intensities in the BSE images because of their similar electron backscattering coefficients (gold: 0.454–0.447 with 0–20% Ag; galena: 0.427) [34, 35], but they could be distinguished in the SE images where galena was darker than gold. The contrast boundaries indicate two minerals with different compositions were in contact, excluding the striped domains with a high BSE intensity within gold grains (red dashed lines, Text B in S1 Text). The EDS analysis indicated that the gold grain had a higher Ag content at the periphery adjacent to opal and a void compared to its interior and the periphery adjacent to sphalerite (Fig 3B). The gold grains was 96% ± 16% Au outside the stripe, 95% ± 14% Au in the region containing the stripe, and 89% ± 12% Au at the periphery where it was visibly enriched with Ag (Fig 3C). The normalized intensity ratio map did not show any patterns correlated with the striped domain (Fig 3D). The absence of compositional variation in the linear domain indicates its crystallographic origin.

The gold grain was surrounded by sphalerite and opal containing voids, and galena was separated from the gold grain by the opal. A void within the gold grain was connected to voids in the surrounding opal (S1 Movie). Particulate matter with a high BSE contrast (23 ± 9 nm, *N* = 7) was observed at the edges of the gold grain near the opal and within the opal, and it was similar in size to those on the unpolished gold grain. However, the NPs were too small for compositional analysis (red triangles in Fig 3A).

**Fig 4:**
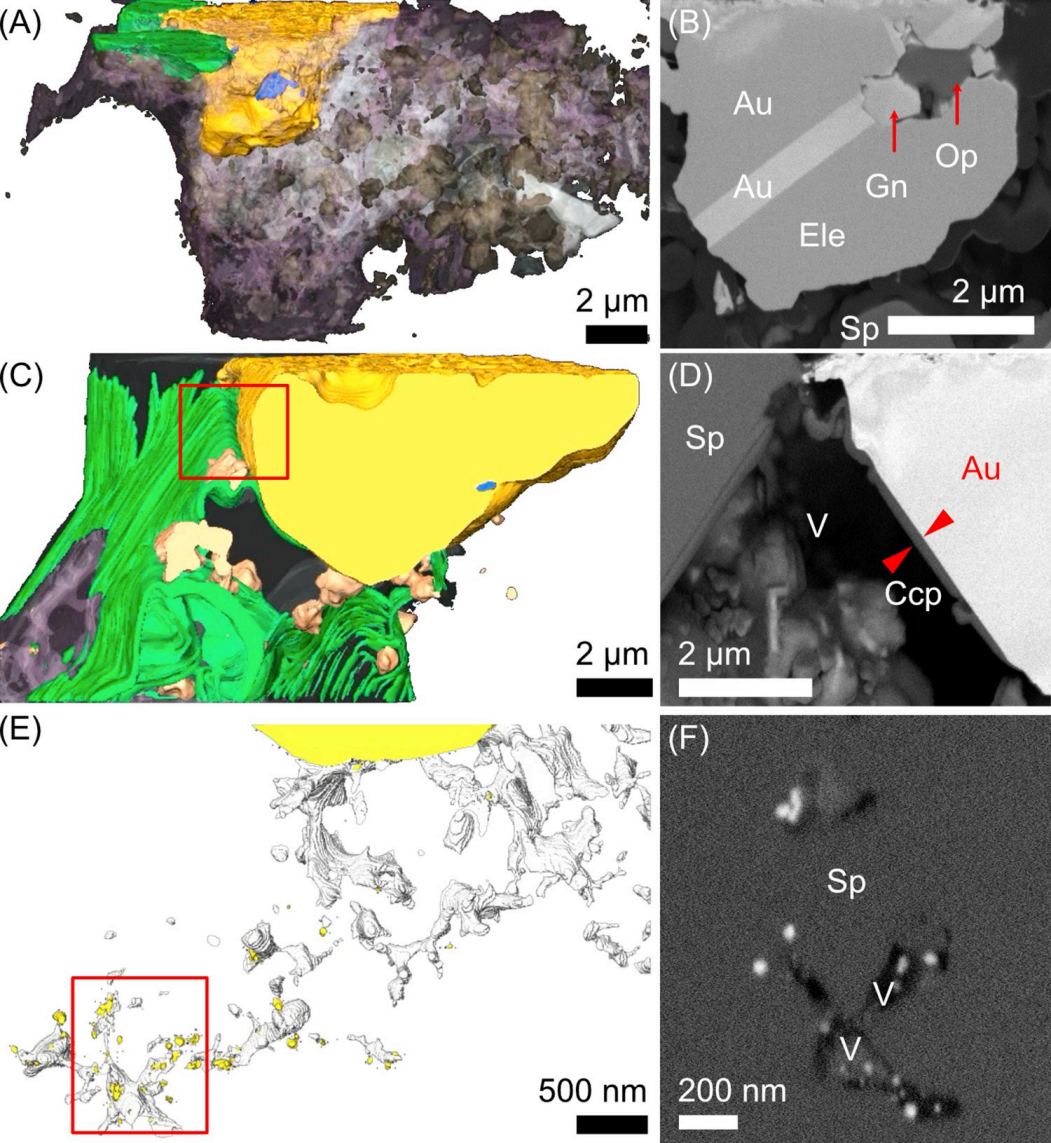
Three-dimensional reconstruction of serial FIB sections. (A) Gold grain shown in Fig 3. (B) Slice of A showing galena surrounded by gold and opal. (C) Gold grain from a dry-polished section. (D) Slice from the red box in C. The red triangles indicate a thin layer of chalcopyrite. (E) Gold grain from a wet-polished section (largest one at the top) and NPs with high BSE contrast that is possibly gold (other than the largest one). (F) Slice from the red box in E showing NPs with a high BSE contrast. Colors: yellow, Au and putative Au (in E); green, Ccp; blue, void on/in gold; purple, Opl; brown, Gn; white, void outside gold. Sphalerite in all images and voids in C are not shown for clarity. Mineral abbreviations are as in Figs 2 and 3. See S1–S3 Movies for further details on Fig 4A, 4C, and 4E, respectively.

Serial sectioning by FIB-SEM and subsequent 3D reconstruction of the gold grain in the dry-polished section provided further insights into the minerals and their formation. The boundaries of sphalerite, galena, and chalcopyrite were sharp while they were less defined for gold and opal, which suggests that the former group precipitated earlier (Fig 4A and S1 Movie). The gold grain contained a galena particle partly surrounded by opal (Fig 4B, Text B in S1 Text), and exhibited randomly oriented and isolated voids with an average spherical diameter of 177 ± 121 nm (*N* = 10, S5 Fig).

In other serial FIB sections of a dry-polished gold grain (Fig 4C and S2 Movie), random patterns in the BSE contrast were observed at the margin down to a depth of 1–3 μm from the adjacent void (S6 Fig). Such a contrast modulation was absent in the gold grain below the polished surface facing the void, which was covered by a ~150 nm-thick chalcopyrite layer (Fig 4D, Text B in S1 Text).

A thick wet-polished section was used to investigate the 3D structure of the gold grains and their surrounding minerals (Fig 4E, S7 Fig, and S3–S8 Movies). Gold grains observed as separate particles were connected inside the thin sections, which indicated that simple counting the number of particles in optical microscopy of the thin sections is not appropriate (Fig E in S7 Fig and S8 Movie). The observed cross-sectional fine features were consistent with those in the dry-polished sections. In particular, NPs (36 ± 12 nm, *N* = 176, S8 Fig) were observed on gold grains near voids or mineral boundaries, and isolated voids were observed within gold grains (S9 Fig). Rod- and plate-shaped particles were also noted that may be clay minerals based on their morphology (S10 Fig). NPs exhibiting a contrast as bright as gold grains were found in voids within sphalerite (22 ± 10 nm diameter, *N* = 53, Fig 4F) that are possibly gold NPs, but they were too small to determine their composition precisely by SEM-EDS.

### Nanoparticles around gold grains

**Fig 5:**
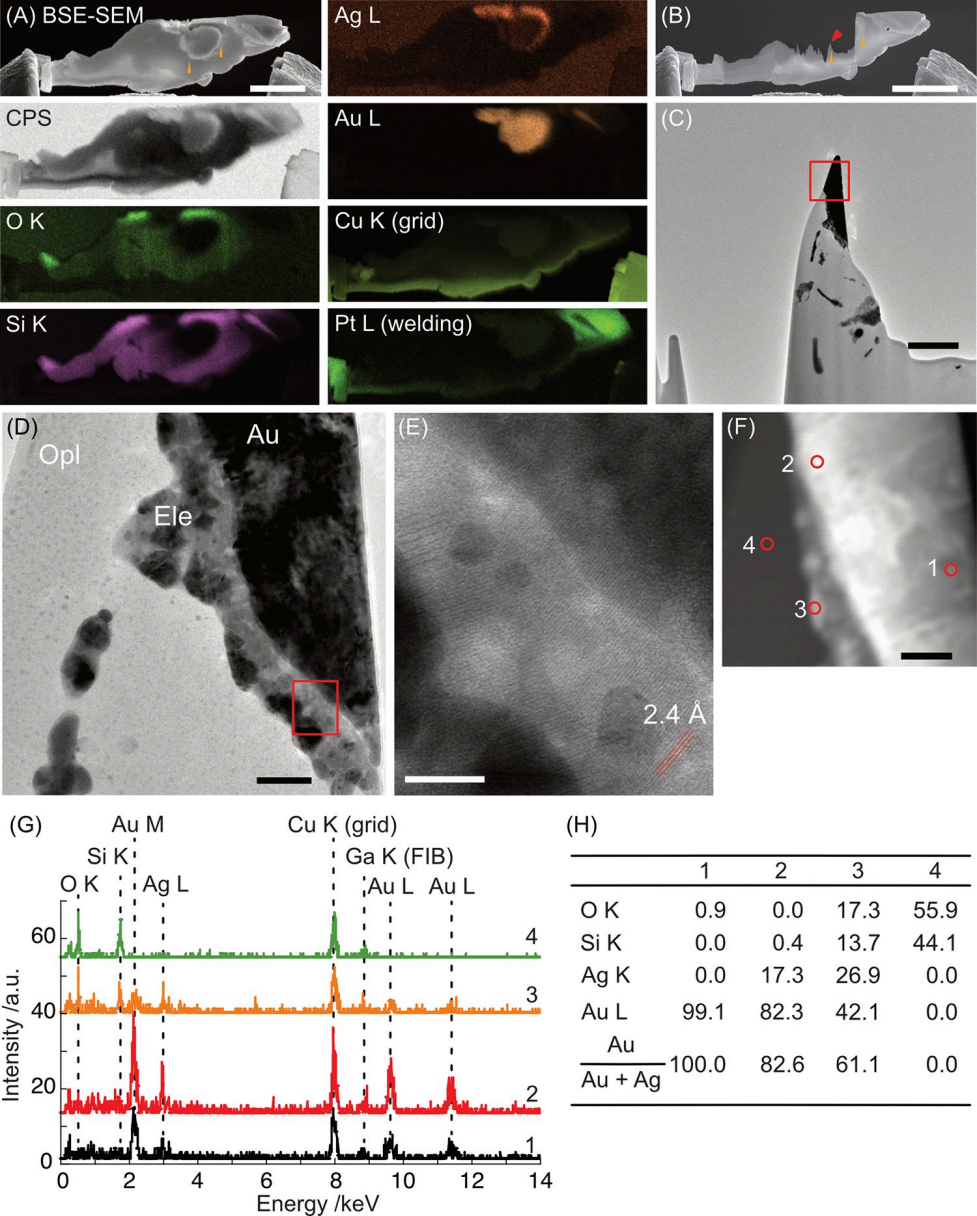
TEM images of a FIB-milled lamella containing an gold grain adjacent to and/or enclosed in opal. (A) SEM/EDS of a lamella picked up using FIB onto a carbon grid before milling. (B) BSE-SEM image of the lamella after milling with a Ga ion beam at 5 kV. Orange triangles indicate the same position as those in A. (C) Low-magnification TEM image of the lamella around the area marked by a red triangle in B. (D) Magnified TEM image of the red squared area in C. (E) Magnification of the red square in D. The red lines indicate a lattice spacing of 2.3–2.4 Å. (F) STEM image of D. (G) STEM/EDS spectra of red circles 1–4 in F. Copper, gallium, and carbon were found as artificial signals originating from the copper grid, ion injection during FIB milling, and residual gas adsorbents, respectively. Note the absence of Pt from welding. (H) Elemental composition (wt%) of points in F. Elements from artificial signals were removed from the analysis. Scale bars: 50 nm in D and F, 10 nm in E. Mineral abbreviations are the same as in Figs 2 and 3 (Ele: electrum).

The detailed structure around the periphery of the gold grains was further investigated by using TEM. A lamella containing a gold grain and adjacent opal identified by SEM-EDS was picked up from a dry-polished section using FIB (Fig 5A) and was further thinned to expose the gold and surrounding minerals (Fig 5B). The TEM images showed the gold grain as dark- and opal as light-contrast objects, and between them was a dark-contrast mineral layer (Fig 5C), comprising NPs (11.9 ± 4.5 nm, *N* = 69, Fig 5D). The lighter contrast of the NP layer relative to the gold grain indicated that the NPs were partly mixed with opal. The NP layer had a straight edge when the NPs were small and a round edge when the NP was larger than ca. 10 nm in diameter. The NPs had a random orientation, and some NPs had a lattice spacing of 0.24 nm, which corresponds to the {111} surface of gold (Fig 5E). STEM/EDS analysis revealed that the gold grain was almost pure gold with a Ag fraction of less than 20% and mineralogically classified as “native gold”, while the surrounding NPs contained up to 61 wt% Ag, corresponding to “electrum NPs” (Fig 5F–5H).

The crystallographic difference between the NPs in and around the gold grains were investigated by selected area electron diffraction (SAED). The SAED pattern indicated that the grain was gold (Fig A and B in S11 Fig). The SAED pattern excluding the gold grain but including the NPs outside the grain edge and surrounding opal indicated that they were also gold with multiple crystalline orientations (Fig C in S11 Fig). Halo peaks from opal were not obvious. Dark-field (DF) TEM images showed that the gold grain comprised small (3.7 ± 1.4 nm, *N* = 199) and large particles (13.0 ± 10.9 nm, *N* = 52, area in green dotted lines), which indicated that the grain comprised NPs aggregated with different crystal orientations and that a portion grew into a large domain (Fig D–F in S11 Fig). The DF-TEM images showed that NPs attached to the grain were bright and formed domains with sizes of 8.4 ± 5.8 nm (*N* = 92, area in blue-dotted area).

## Discussion

The mound rock sample at the HAKC HF was studied by preparing three types of sections: thin sections prepared by wet and dry polishing and cross-sections prepared by FIB milling. In the FIB-milled cross-section, voids were clearly observed around gold adjacent to sphalerite, galena, and opal. These voids were present in the dry-polished thin sections and absent in the wet-polished sections, which indicated that the voids were inherent to the sample. Localized Ag features were also observed only in the dry-polished and FIB-milled sections. The absence of features in the wet-polished sections can be attributed to the high ductility of gold; this polishing method induces shear forces that restructured the gold grains to cover adjacent voids and normalized the Ag intensity.

The morphology and type of surrounding or included minerals and voids provide insights into the gold formation process. The presence of galena inside gold grains suggests that the former formed prior to the precipitation of the latter (Fig 2A). The presence of opal as partially incorporated within gold but primarily external indicates that it precipitated at a temperature similar to or lower than that of gold. Opal was also observed adjacent to voids that were interconnected through large voids underlying the polished surface, submicron channels, and the grain boundaries of minerals. Gold facing the voids were in contact with opal, but in one case a thin chalcopyrite layer formed on the gold, which was assumed to be redeposition after the precipitation of gold (Fig 4C and 4D). Because chalcopyrite typically precipitates at temperatures of 280–320°C or lower [36], the observed voids represent the paths of hydrothermal fluid, and the fluid temperature may have increased to precipitate chalcopyrite after gold precipitated at a lower temperature. The selected mound rock sample was exposed on the seafloor for a long time and would have undergone overprinting mineralization by subsequent hydrothermal activity, mineral replacement, and seafloor weathering.

NPs with a size of 5–50 nm in size were identified on and around the gold grains in both a serial FIB-SEM cross-sections and in a TEM lamella. The lattice spacing, elemental composition, and DF-TEM images indicated that the NPs on and in the gold grains were mostly single-crystal electrum containing a significantly higher Ag content than the large gold grains. The NP size was consistent with the previous reports of gold and electrum NPs in hydrothermal fluids with a size of <10 nm [10, 13, 14, 16], which grow to ~20 nm or more by Ostwald ripening [9, 10] as observed on quartz and in quartz fluid inclusions [11, 12].

The DF-TEM images showed that the gold grain were smaller than the attached NPs, which implies that the gold grain did not grow simply by the coalescence of electrum NPs but either by recrystallization from nanoscale electrum melts [37] or by a ripening processes. The decreased atomic fraction of Ag from NPs attached to the gold grain to within the gold grain center indicates the partial removal of Ag during the merging process. This may be because Ag is more soluble than Au in sulfidic hydrothermal fluid by one to three orders of magnitude [7]. The removal of Ag from the gold grains would have eventually caused shrinkage of the particle size. The removed Ag then precipitate as Ag-bearing minerals within opal such as pyrargyrite and acanthite (S2 Fig) while some Ag remained in gold around the grain surface because it is thermodynamically favored to be at the surface of an Ag–Au cluster [38]. This process may have taken place at relatively low temperatures of approximately 100°C considering the low melting point of smaller gold NPs [39] and the surrounding opal as a matrix of attached electrum NPs. Long after the incorporation of NPs, thermal recrystallization may have taken place that reordered gold atoms to form larger single-crystal domains (Fig C and D in S11 Fig).

In summary, gold and electrum NPs that were 5–50 nm in size were found attached on gold grains and in the surrounding voids, which can be associated with the “invisible” gold NPs of hydrothermal fluid. The dry-polishing method minimized damage to sample, and the FIB-SEM method exposed samples that has not mechanically damaged, which facilitated visualization of the evolution of NPs within the minerals. Ag that was initially incorporated in the electrum NPs was then partly removed during mineral growth. Some of the gold NPs were involved in the formation of larger gold grains. The gold grains were nonuniform in composition, and Ag was preferentially localized on their surface indicating post-deposition or secondary remobilization. Because the formation of NPs depends on the supersaturation concentration of Au, further investigation is needed to determine whether a similar mechanism of gold grain formation applies to hydrothermal sites with a lower Au content or a different fluid chemistry [20]. In addition, the initial stages of gold grain formation can be more clearly visualized from samples obtained through seafloor drilling, which would include rocks closely associated with subseafloor boiling [13, 14].

## Supporting information

S1 Fig Microscopy images showing mineral identification and occurrence of gold in the wet-polished section under the microscope. (A) Gold at the edge of sphalerite. (B) Gold in the periphery of sphalerite and in contact with a silica mineral (opal). Gold is are marked by red triangles. Abbreviations: Au, native gold; Brt, barite; Ccp, chalcopyrite; Gn, galena; Opl, opal; Py, pyrite; Sp, sphalerite; V, void.
(TIF)

S2 Fig Constituent minerals in wet-polished sections including an Ag-rich mineral. (A) Reflected-light photomicrograph. (B) BSE image of the red squared part in (A). (C) EDS elemental maps of (B). Acanthite (Aca) indicated by red arrows in B and C. Abbreviations are the same as in S1 Fig.
(TIF)

S3 Fig Dry-polished section showing a gold grain occurring within a void. (A) SE-SEM image of a gold grain surrounded by a void. (B) Magnification of the red squared area in A. (C) EDS elemental map of the full area of A. The aluminum-rich domain is marked by red triangles in A and C, which was aggregated alumina abrasive with attached cutting dusts. Abbreviations are the same as in S1 Fig.
(TIF)

S4 Fig SEM-EDS analysis of abrasive residue in voids next to a electrum grain. (A) SEM image, (B) EDS elemental maps, and (C) elemental composition (wt%) of the red square in A.
(TIF)

S5 Fig Comparison of SEM images and isolated voids. (A) SE and (B) BSE images of a gold grain and surrounding minerals within a dry-polished section at a depth of 1.0 μm from the section in Fig 3. Minerals were assigned based on the cross-sectional EDS maps in Fig 3. (C) Qualitative comparison of the relative contrasts of four major minerals in the SE and BSE images. (D) Isolated voids observed within the gold grain of the dry-polished section in Fig 4A. Abbreviations are the same as in S1 Fig.
(TIF)

S6 Fig Depth-dependent contrast change within a gold grain in a dry-polished section. The depth from the surface is denoted by yellow letters, and the contrast modulation around the polished surface is shown by red dotted lines. Abbreviations are the same as in S1 Fig.
(TIF)

S7 Fig Three-dimensional reconstruction of a wet-polished sections. (A–E) Snapshots from S3–S7 Movies. Minerals are coded by color: gold (bright yellow), galena (pale orange), chalcopyrite (green), voids (white), and voids next to gold (blue). To facilitate visualization, opal and sphalerite are not shown.
(TIF)

S8 Fig Electrum nanoparticles of a wet-polished section attached to a gold grain. (A) Slice from S7A Fig. (B) Magnification of the red square in A. (C) Slice from S7B Fig. (D) Magnification of the red square in C. Abbreviations are the same as in S1 Fig.
(TIF)

S9 Fig Isolated void within a gold grain of a wet-polished section. (A) Slice from S7C Fig. (B) Depth-dependent high-magnification images showing the isolated void within the gold grain indicated by the red square in A. Abbreviations are the same as in S1 Fig.
(TIF)

S10 Fig Rod- and plate-shaped particles on a gold grain in contact with a vacancy in the wet-polished FIB cross section. (A) Slice from S7D Fig. (B) Magnification of the red square in A. The rod-shaped particles are indicated by red rectangles. Abbreviations are the same as in S1 Fig.
(TIF)

S11 Fig Dark-field (DF) TEM image of the FIB-milled lamella in Fig 5. (A) Bright-field image. SAED patterns of the (B) red circle and (C) yellow circle in A. (D) Diffraction pattern of the full area. DF-TEM images from the diffraction spots of the (E) red circle and (F) yellow circle in (D). Green and blue-dotted lines were added to facilitate comparison of the overall shapes of the gold grain and NPs at the periphery, respectively. Scale bars: 100 nm.
(TIF)

S1 Movie SEM images of serial sections and 3D reconstruction of the dry-polished gold grain in Fig 4A. The movie includes BSE images followed by 3D reconstruction. Colors are the same in Fig 4 except for sphalerite (transparent blue green). Unless otherwise noted, other movies have the same structure and coloring. Field of view is 25.3 μm × 14.0 μm × 21.6 μm.
(MOV)

S2 Movie SEM images of serial sections and 3D reconstruction of the dry-polished gold grain in Fig 4C. Field of view is 25.0 μm × 9.2 μm × 7.9 μm. See S1 Movie captions for other information.
(MOV)

S3 Movie SEM images of serial sections and 3D reconstruction of the wet-polished gold grain in Fig 4E. Field of view is 6.3 μm × 4.2 μm × 1.5 μm. See S1 Movie captions for other information.
(MOV)

S4 Movie SEM images of serial sections and 3D reconstruction of the wet-polished gold grain in S7A Fig. SE serial section images are added in the beginning. Field of view is 19.5 μm ×13.0 μm ×6.6 μm. See S1 Movie captions for other information.
(MOV)

S5 Movie SEM images of serial sections and 3D reconstruction of the wet-polished gold grain in S7B Fig. Field of view is 7.3 μm × 5.0 μm × 3.2 μm. See S1 Movie captions for other information.
(MOV)

S6 Movie SEM images of serial sections and 3D reconstruction of the wet-polished gold grain in S7C Fig. Field of view is 25.4 μm × 16.9 μm × 8.7 μm. See S1 Movie captions for other information.
(MOV)

S7 Movie SEM images of serial sections and 3D reconstruction of the wet-polished gold grain in S7D Fig. Field of view is 15.9 μm × 10.6 μm × 9.6 μm. Sphalerite was not reconstructed to facilitate visualization.
(MOV)

S8 Movie SEM images of serial sections and 3D reconstruction of the wet-polished gold grain in S7E Fig. Field of view is 25.4 μm × 16.9 μm × 9.7 μm. Sphalerite was not reconstructed to facilitate visualization.
(MOV)

S1 Text Supplementary discussion on sample damage and image interpretation. (A) Cause and morphology of surface artifacts on polished gold grains. (B) Origin of contrast deviations within cross-sectional SEM images of gold grains. Supplementary references are included.
(DOCX)

S1 Table Elemental composition of representative minerals (wt%, atom%) quantified by EPMA. (XLSX)
