# Supplementary figures and images for: Microscopic evidence for nanoparticle-mediated growth of native gold in sulfide deposits at the Higashi–Aogashima Knoll Caldera hydrothermal field
Source: PLoS One. 2025 Jan 17;20(1):e0317220. doi: 10.1371/journal.pone.0317220 (PMC11741634; doi:10.1371/journal.pone.0317220)

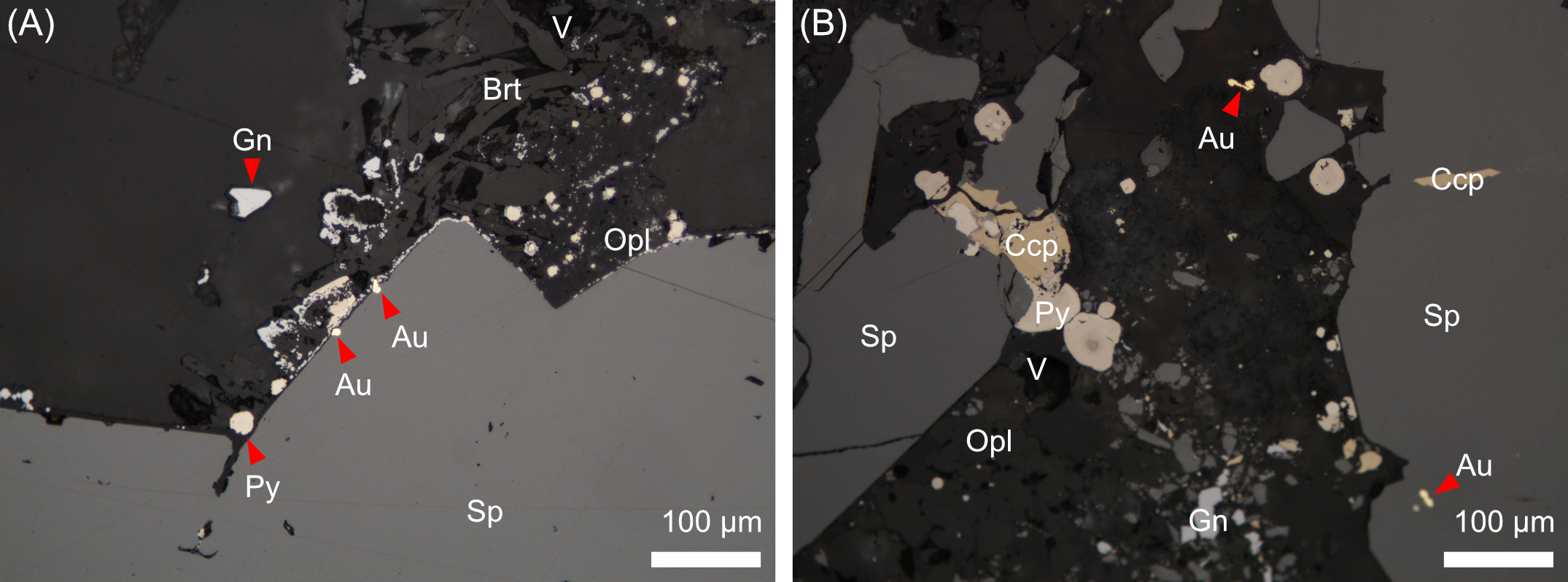

Supplement: S1 Fig — (A) Gold at the edge of sphalerite. (B) Gold in the periphery of sphalerite and in contact with a silica mineral (opal). Gold is are marked by red triangles. Abbreviations: Au, native gold; Brt, barite; Ccp, chalcopyrite; Gn, galena; Opl, opal; Py, pyrite; Sp, sphalerite; V, void. (TIF) [file pone.0317220.s001.tif]

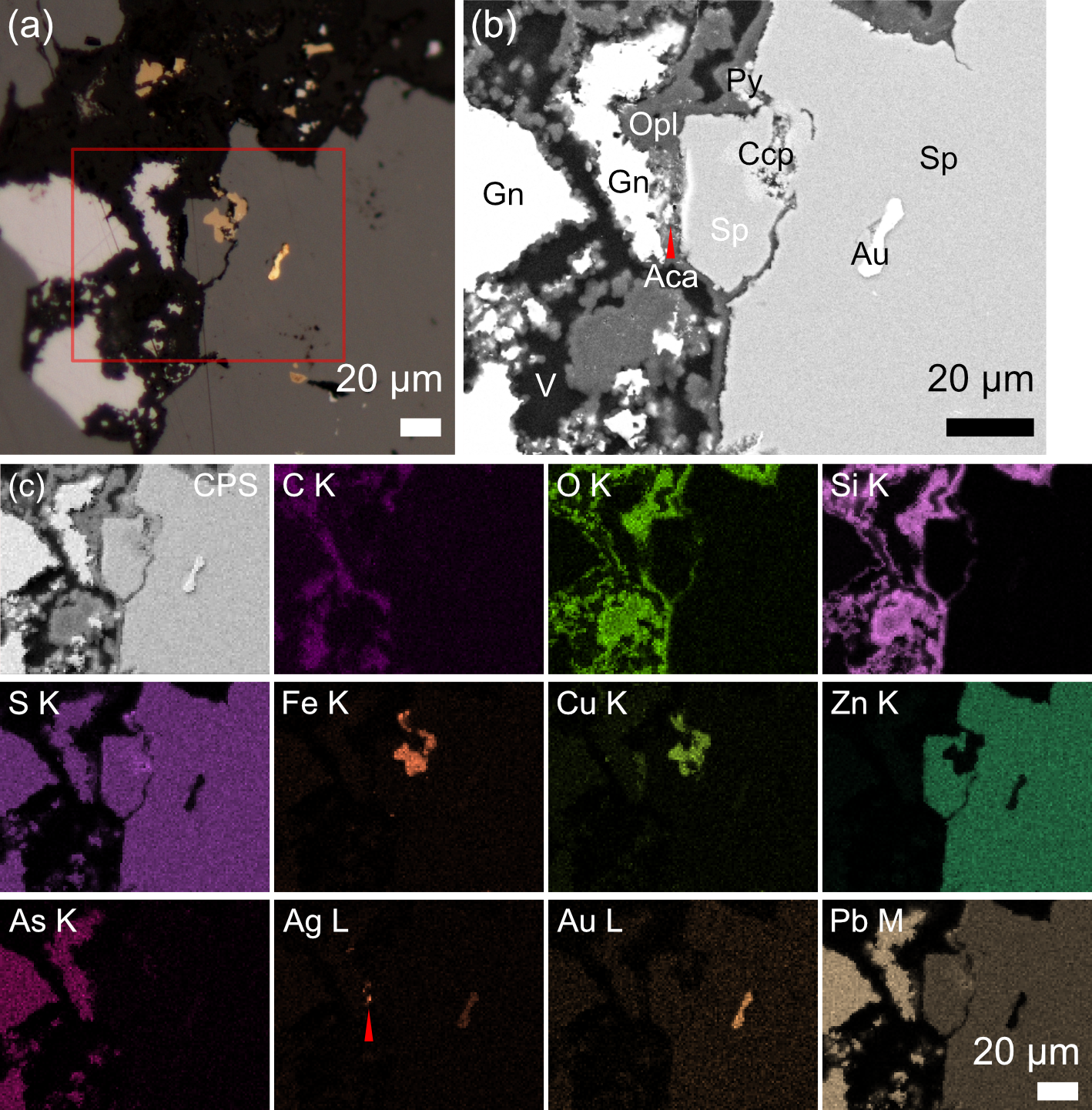

Supplement: S2 Fig — (A) Reflected-light photomicrograph. (B) BSE image of the red squared part in (A). (C) EDS elemental maps of (B). Acanthite (Aca) indicated by red arrows in B and C. Abbreviations are the same as in S1 Fig. (TIF) [file pone.0317220.s002.tif]

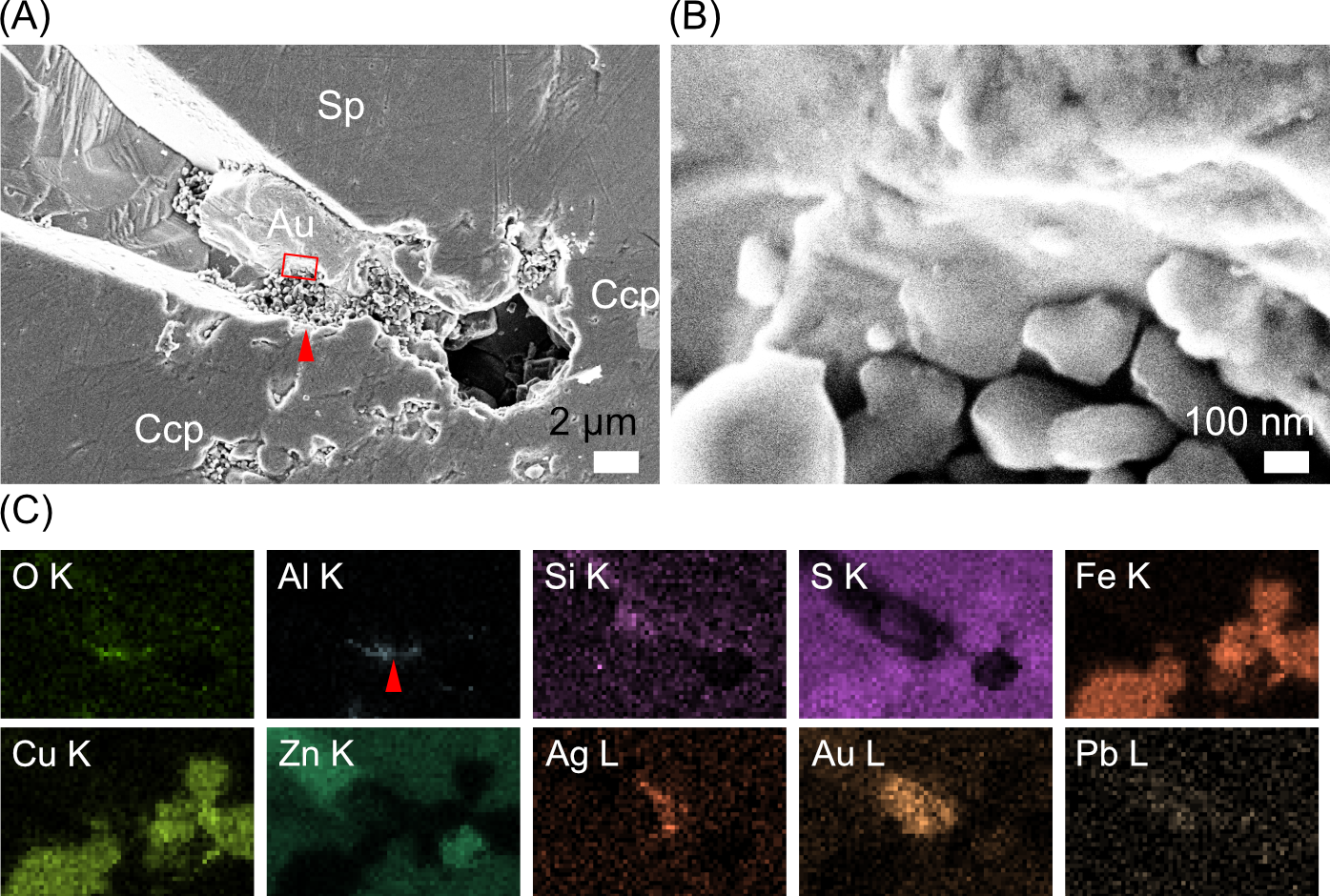

Supplement: S3 Fig — (A) SE-SEM image of a gold grain surrounded by a void. (B) Magnification of the red squared area in A. (C) EDS elemental map of the full area of A. The aluminum-rich domain is marked by red triangles in A and C, which was aggregated alumina abrasive with attached cutting dusts. Abbreviations are the same as in S1 Fig. (TIF) [file pone.0317220.s003.tif]

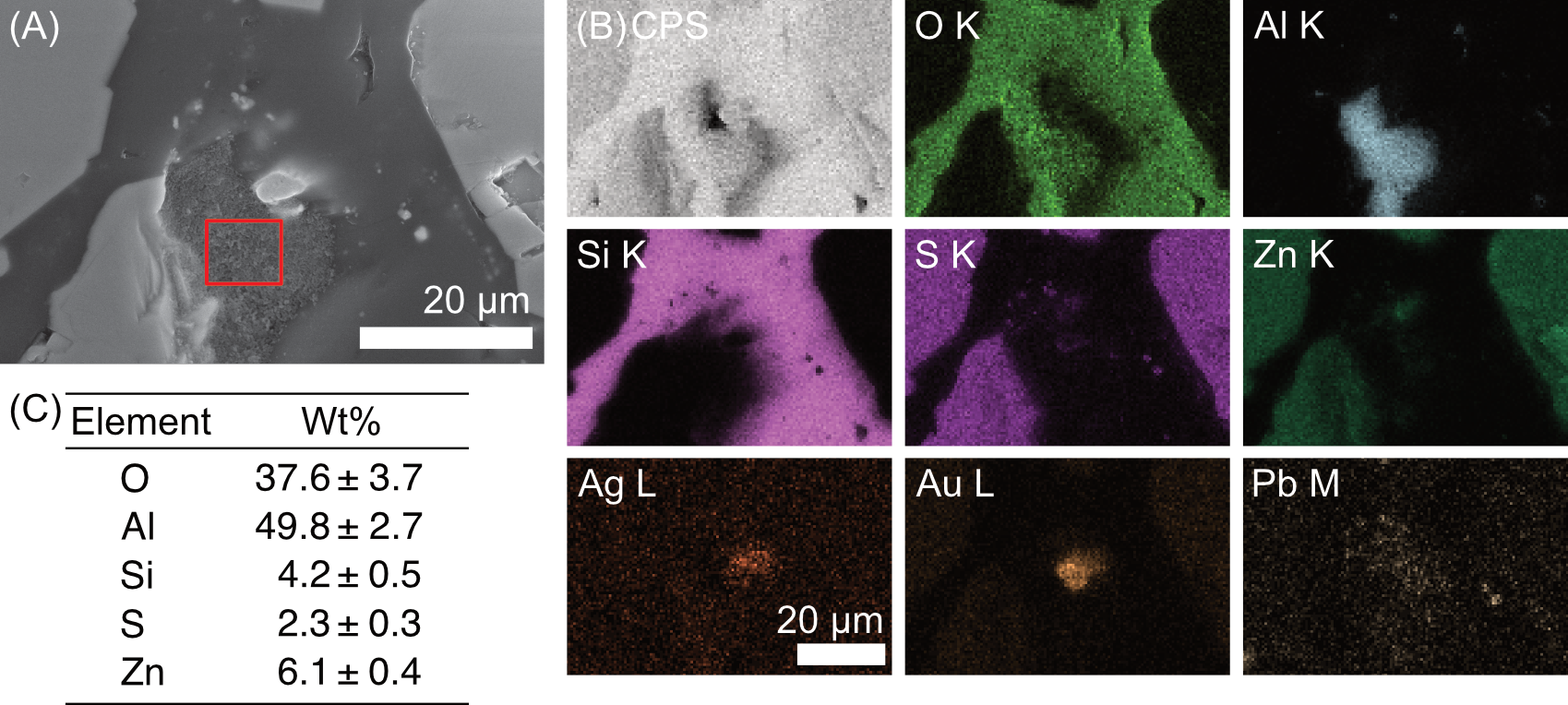

Supplement: S4 Fig — (A) SEM image, (B) EDS elemental maps, and (C) elemental composition (wt%) of the red square in A. (TIF) [file pone.0317220.s004.tif]

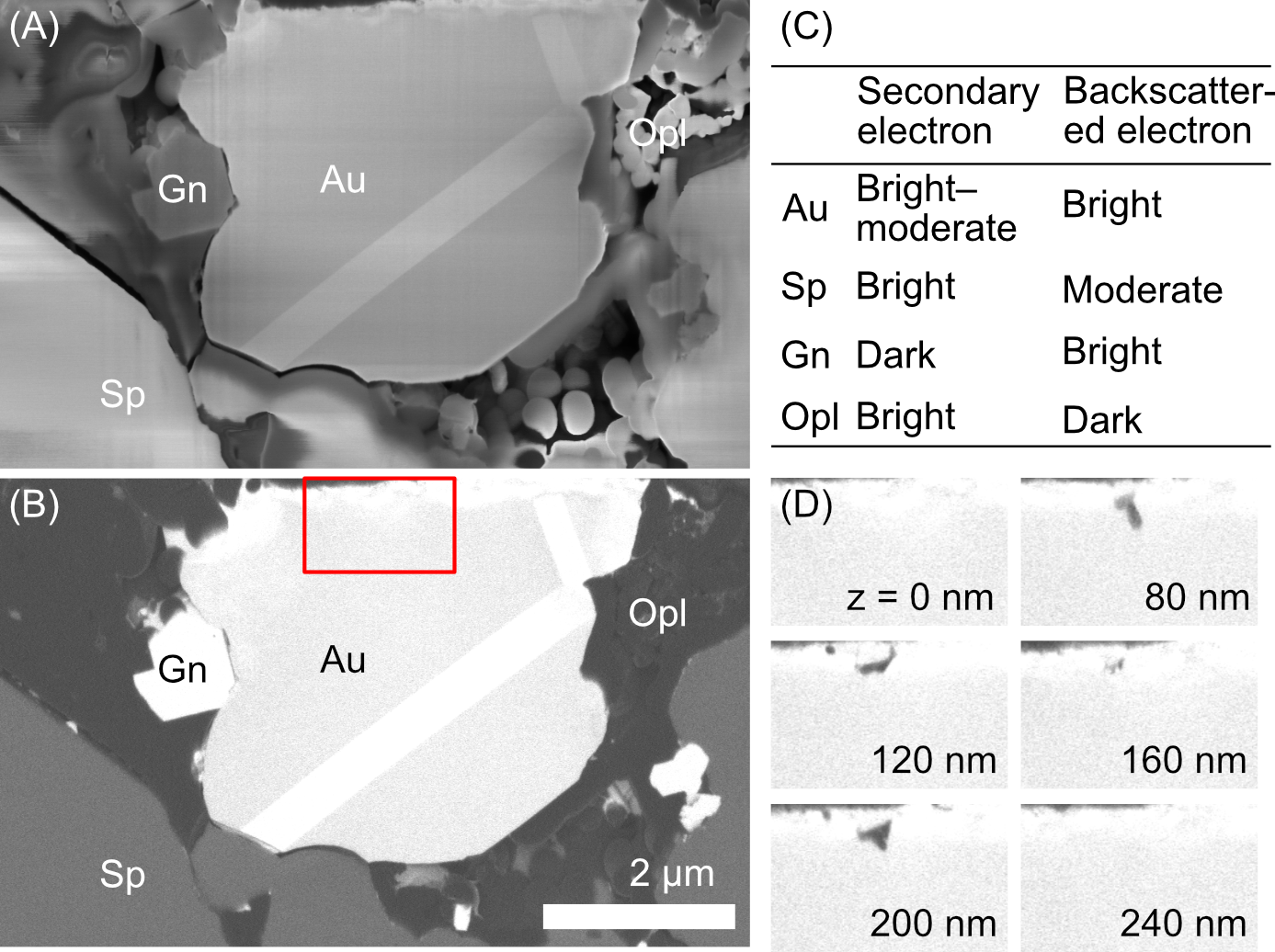

Supplement: S5 Fig — (A) SE and (B) BSE images of a gold grain and surrounding minerals within a dry-polished section at a depth of 1.0 μm from the section in Fig 3. Minerals were assigned based on the cross-sectional EDS maps in Fig 3. (C) Qualitative comparison of the relative contrasts of four major minerals in the SE and BSE images. (D) Isolated voids observed within the gold grain of the dry-polished section in Fig 4A. Abbreviations are the same as in S1 Fig. (TIF) [file pone.0317220.s005.tif]

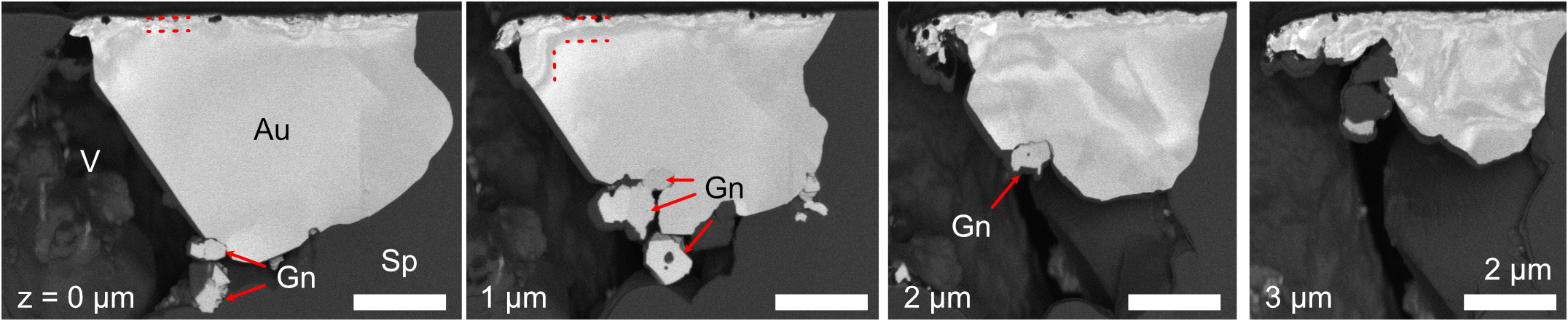

Supplement: S6 Fig — The depth from the surface is denoted by yellow letters, and the contrast modulation around the polished surface is shown by red dotted lines. Abbreviations are the same as in S1 Fig. (TIF) [file pone.0317220.s006.tif]

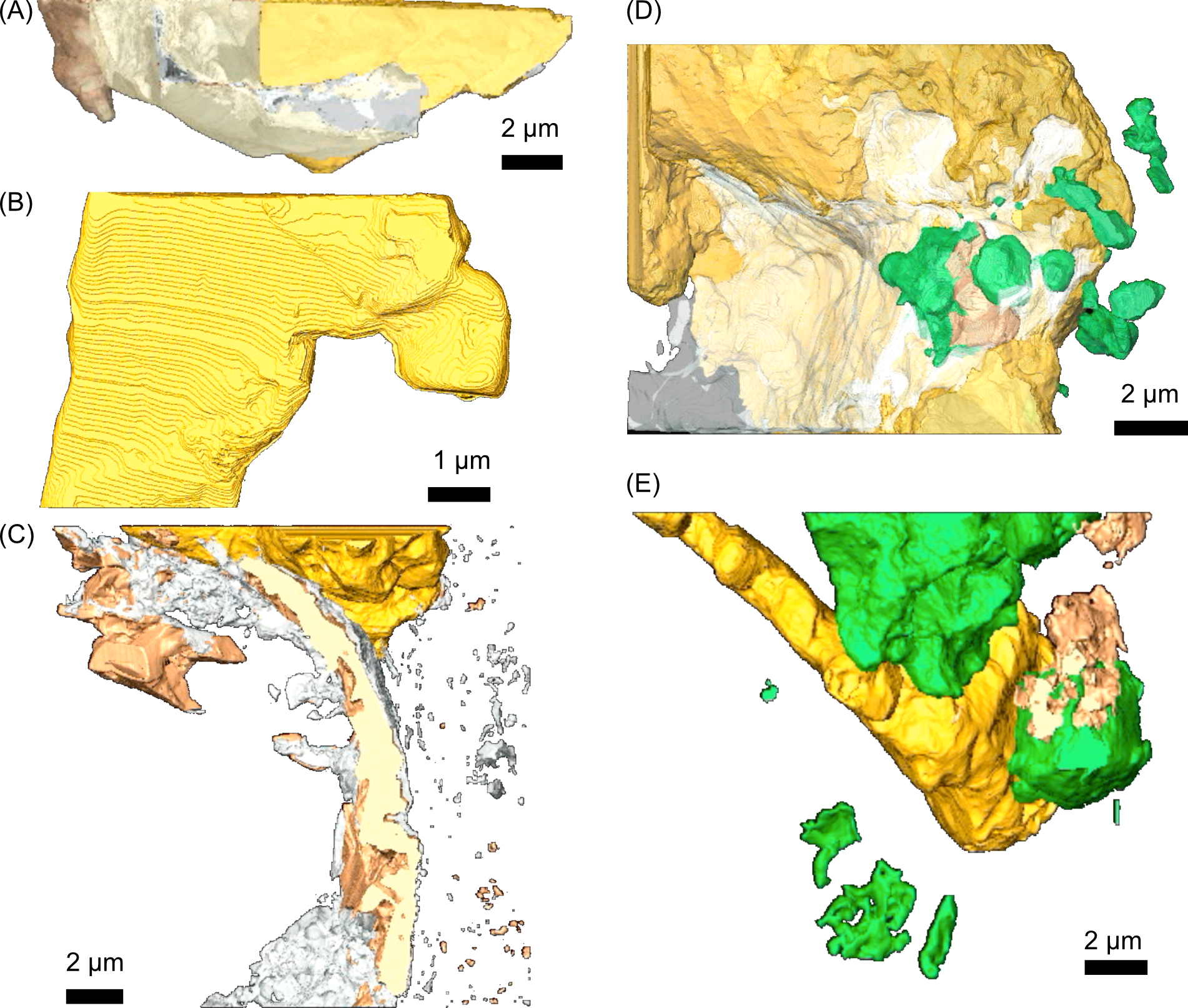

Supplement: S7 Fig — (A–E) Snapshots from S3–S7 Movies. Minerals are coded by color: gold (bright yellow), galena (pale orange), chalcopyrite (green), voids (white), and voids next to gold (blue). To facilitate visualization, opal and sphalerite are not shown. (TIF) [file pone.0317220.s007.tif]

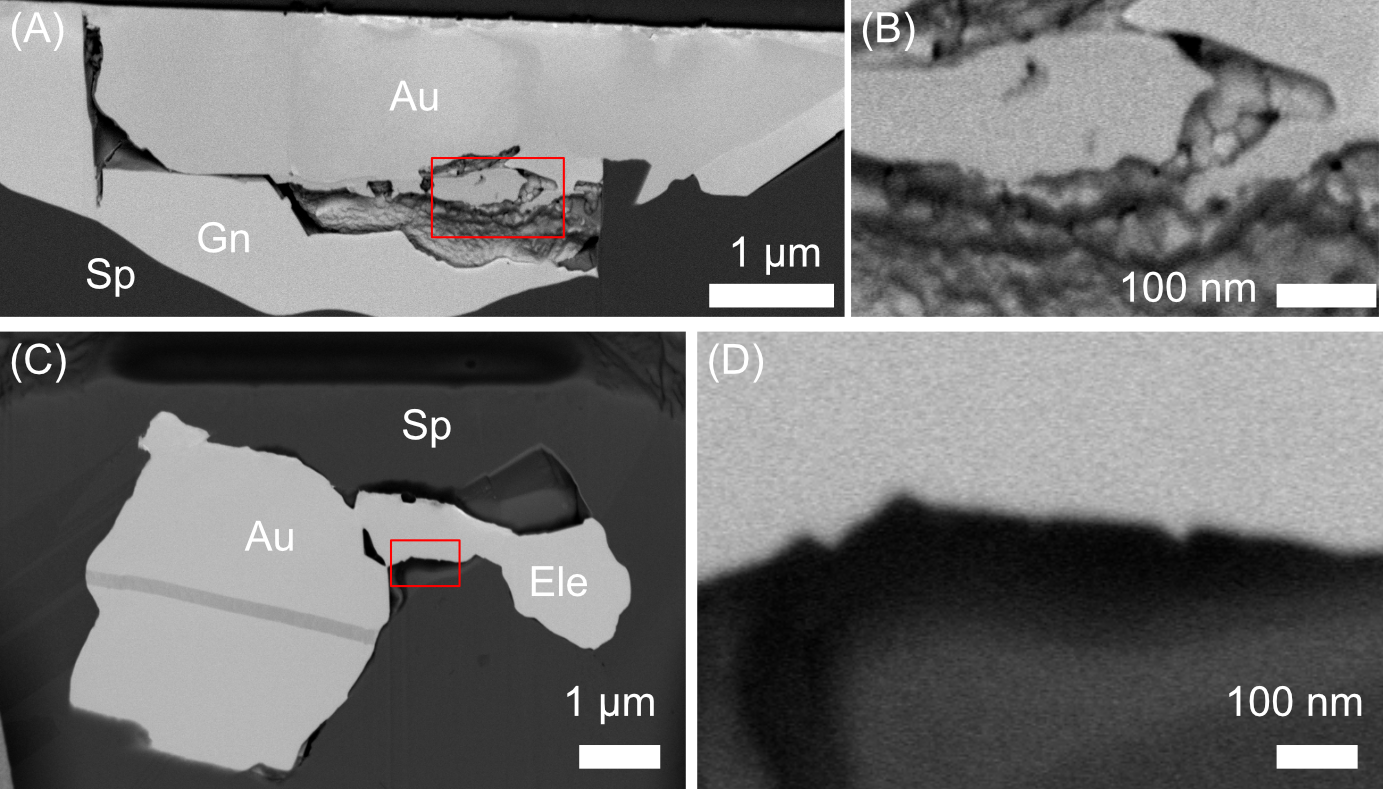

Supplement: S8 Fig — (A) Slice from S7A Fig. (B) Magnification of the red square in A. (C) Slice from S7B Fig. (D) Magnification of the red square in C. Abbreviations are the same as in S1 Fig. (TIF) [file pone.0317220.s008.tif]

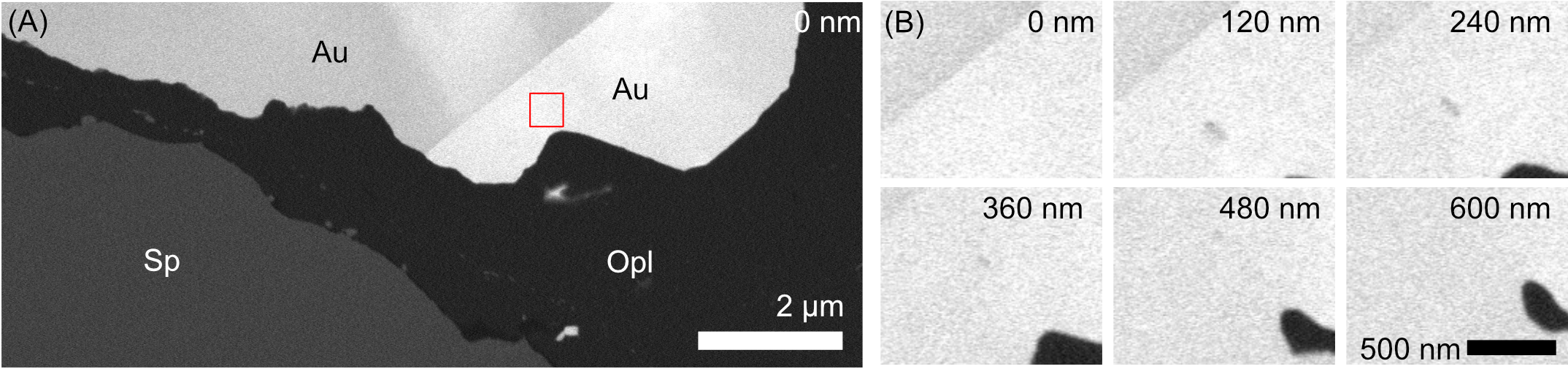

Supplement: S9 Fig — (A) Slice from S7C Fig. (B) Depth-dependent high-magnification images showing the isolated void within the gold grain indicated by the red square in A. Abbreviations are the same as in S1 Fig. (TIF) [file pone.0317220.s009.tif]

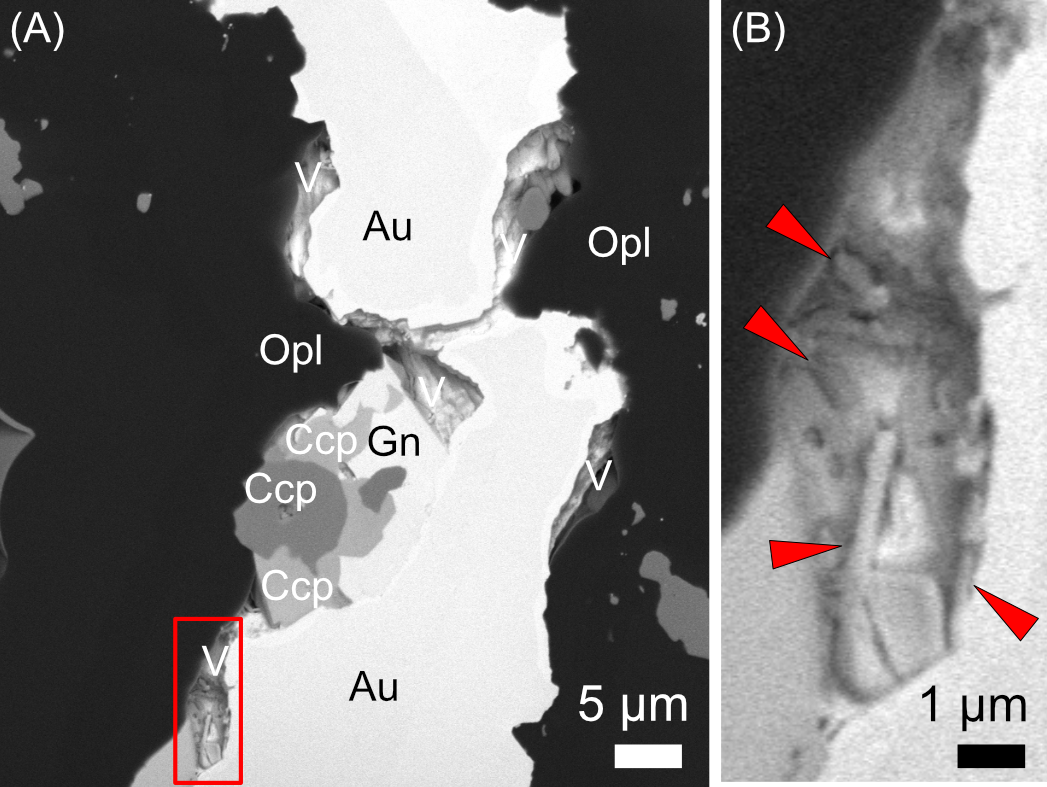

Supplement: S10 Fig — (A) Slice from S7D Fig. (B) Magnification of the red square in A. The rod-shaped particles are indicated by red rectangles. Abbreviations are the same as in S1 Fig. (TIF) [file pone.0317220.s010.tif]

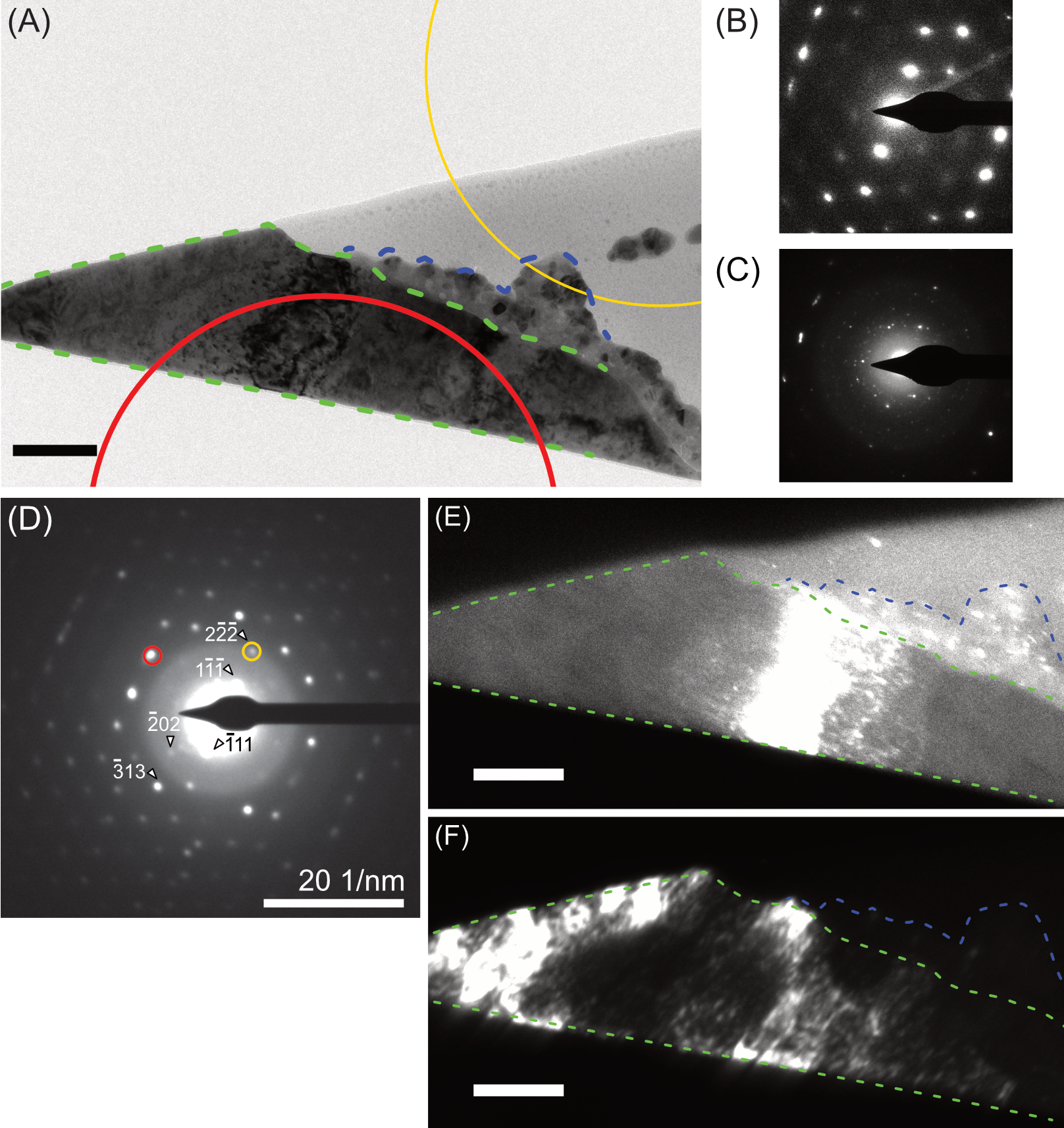

Supplement: S11 Fig — (A) Bright-field image. SAED patterns of the (B) red circle and (C) yellow circle in A. (D) Diffraction pattern of the full area. DF-TEM images from the diffraction spots of the (E) red circle and (F) yellow circle in (D). Green and blue-dotted lines were added to facilitate comparison of the overall shapes of the gold grain and NPs at the periphery, respectively. Scale bars: 100 nm. (TIF) [file pone.0317220.s011.tif]
