# Supplementary material for: Microscopic evidence for nanoparticle-mediated growth of native gold in sulfide deposits at the Higashi–Aogashima Knoll Caldera hydrothermal field
Source: PLoS One. 2025 Jan 17;20(1):e0317220. doi: 10.1371/journal.pone.0317220 (PMC11741634; doi:10.1371/journal.pone.0317220)
Supplement: S1 Text — (A) Cause and morphology of surface artifacts on polished gold grains. (B) Origin of contrast deviations within cross-sectional SEM images of gold grains. Supplementary references are included. (DOCX) [file pone.0317220.s020.docx]

# Supplementary discussion for

Microscopic evidence for nanoparticle-mediated growth of native gold in sulfide deposits at the Higashi–Aogashima Knoll Caldera hydrothermal field

## Damage and artifact during polishing

There were ~100 nm-sized dark-contrast holes in the top-view SEM image of the dry-polished electrum grains (Figs 2F and S2D Fig), which is also noticeable under stereomicroscope (Fig 2D), while they were absent in wet-polished grains. We assume that most of these holes are artifacts during sample preparation due to the following reasons. The 3D reconstruction showed that these holes are intensively localized only at the polished surface (S1 Movie). If these holes are formed during the formation of the electrum grain, the number and density of these holes should be similar at apart from the polished surface. One possible cause of these holes is the pushing of the alumina abrasive powder that slightly deformed the electrum surface.

Dry polishing is performed without any lubricants that wash away abrasive dusts, and there is a possibility for these dusts and the abrasive powder to be trapped within the rough surface of the mineral such as voids. The regular-shaped angular particles within the electrum grain (231 ± 85 nm in diameter, *N* = 299, S2A–S2C Figs) were often found within the voids. Their elemental composition by EDS analyses were Al : O = 1 : 1.5, showing that they alumina abrasive (Al_2_O_3_). We also found attached smaller particles having similar BSE intensity to that of underlying electrum (33 ± 10 nm in diameter, *N* = 61, S2B Fig). Because these nanoparticles were found on the abrasive as well as on the surface of electrum grains and do not appear to be partly fused to the electrum grain, they are highly possible to be derived from cutting dusts of the specimen minerals. Despite these debris, the dry polishing method enabled us to visualize voids neighboring electrum that cannot be observed in conventional wet polishing owing to the ductile deformation of electrum. Note that voids in the FIB cross-sections below surface is not affected by the polishing methods, because the sample damage caused by polishing is less than hundreds of nanometers depth, as discussed below.

## The contrast deviation within an electrum grain

The contrast deviation observed in the BSE images of Fig. 3A and S6 are attributable to the electron channeling caused by the difference in crystal orientation along with the beam axis [1,2]. Straight patterns were observed in Figs 3A and 4B and S6A Fig (from the same electrum grain, S1 Movie), and S2–S9 Movies (from different grains), indicating the universal occurrences of recrystallization and twinning after overgrowth of electrum grains. In contrast, the random BSE contrast was only observed at the latter part of S6 Fig (S2 Movie), at the beginning of S6 Movie, and at the middle of S7 Movie. All of them are in contact with vacancy whose size is ranging from < 1 to ~10 μm, but the reverse trend is not always true (e. g. S5 and S8 Movies). These observations suggest that the contrast-modulated domain was formed at the later stage of electrum precipitation by cooling and accumulation of small electrum nanoparticles. After electrum deposition, the disordered domain did not undergo extensive heating or dissolution.

There was a thin layer of the contrast-modulated domain around the polished surface (S6 Fig). The domain thickness was 249 ± 54 nm and 207 ± 60 nm for wet-polished sections and dry-polished thin sections, respectively. There are two possible damage sources: gallium ion penetration during FIB-SEM imaging and mechanical shear stress during the polishing. Gallium ion can penetrate gold in the depth of ~10 nm to change crystal orientation and structure [3,4], and it is not deep enough to explain all the damage layer. The subsurface damage by mechanical polishing depends on the diameter of the abrasive powder, and can cause ~200 nm damage when using 1 μm diamond abrasive powder to GaN [5] and ~2 μm damage after polishing ductile Pd foil with 8 μm emery paper [6]. These examples strongly suggest that the origin of the damaged layer is derived from polishing, and the crystallographic damage is inevitable even when using the dry-polishing method.

# Supplementary References

1. Coates DG. Kikuchi-like reflection patterns obtained with the scanning electron microscope. Philos Mag: A J Theor Exp Appl Phys. 1967;16: 1179–1184. doi:10.1080/14786436708229968

2. Booker GR, Shaw AMB, Whelan MJ, Hirsch PB. Some comments on the interpretation of the ‘kikuchi-like reflection patterns’ observed by scanning electron microscopy. Philos Mag: A J Theor Exp Appl Phys. 1967;16: 1185–1191. doi:10.1080/14786436708229969

3. Babu RP, Irukuvarghula S, Harte A, Preuss M. Nature of gallium focused ion beam induced phase transformation in 316L austenitic stainless steel. Acta Mater. 2016;120: 391–402. doi:10.1016/j.actamat.2016.08.008

4. Prenitzer BI, Urbanik-Shannon CA, Giannuzzi LA, Brown SR, Irwin RB, Shofner TL, et al. The Correlation between Ion Beam/Material Interactions and Practical FIB Specimen Preparation. Microsc Microanal. 2003;9: 216–236. doi:10.1017/s1431927603030034

5. Lee J, Kim JC, Kim J, Singh RK, Arjunan AC, Lee H. Evaluation of subsurface damage inherent to polished GaN substrates using depth-resolved cathodoluminescence spectroscopy. Thin Solid Films. 2018;660: 516–520. doi:10.1016/j.tsf.2018.07.002

6. Murase Y, Miyauchi N, Itakura A, Katayama H. Evaluation of Surface Damage of Pd Using Cross-Sectional Electron Backscatter Diffraction Analysis. Mater Trans. 2021;62: 41–47. doi:10.2320/matertrans.mt-m2020220
